# Supplementary material for: Humidity-driven ABA depletion determines plant-pathogen competition for leaf water
Source: Nat Commun. 2025 Dec 19;17:787. doi: 10.1038/s41467-025-67469-y (PMC12824301; doi:10.1038/s41467-025-67469-y)
Supplement: Supplementary file 1 — Supplementary Information [file 41467_2025_67469_MOESM1_ESM.pdf]

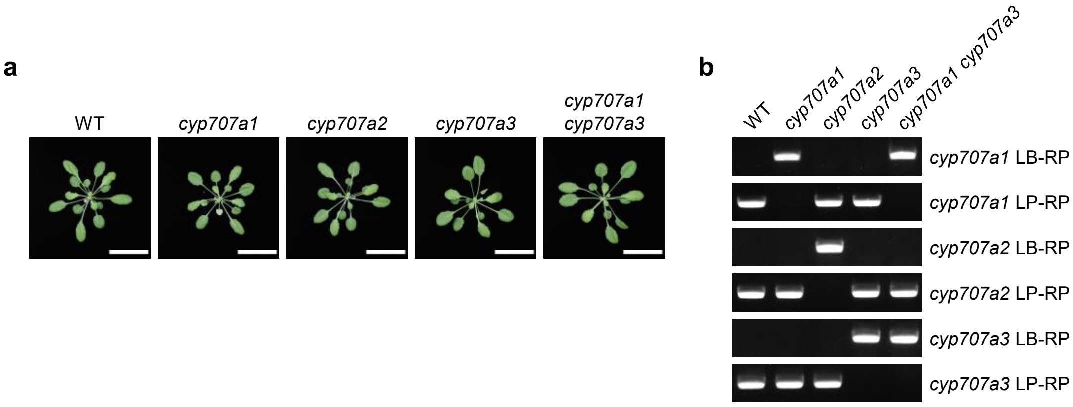

Supplementary Fig. 1: Characterization of *cyp707a* mutant plants.
a, Five-week-old plants grown under short-day conditions. Scale bars = 3 cm. **b**, Genotyping of the indicated plants by PCR. LB-RP and LP-RP primer pairs specifically amplify mutated and non-mutated DNA, respectively.

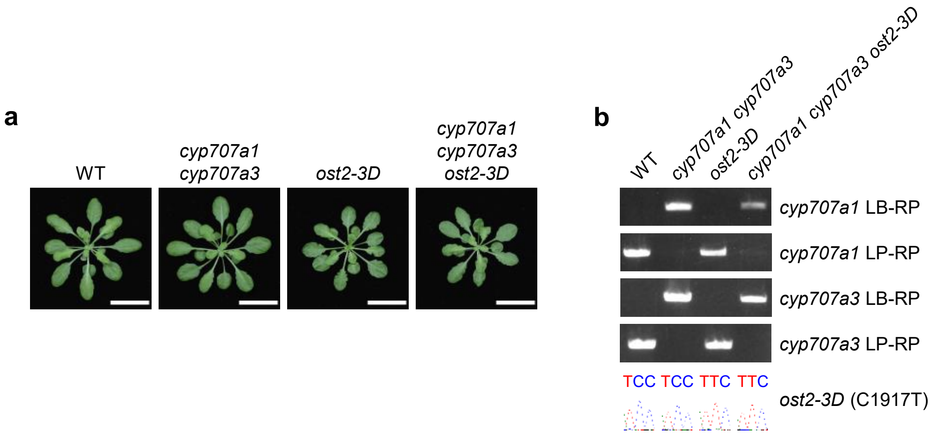

Supplementary Fig. 2: Characterization of *cyp707a1 cyp707a3 ost2-3D* mutant plants.
a, Five-week-old plants grown under short-day conditions. Scale bars = 3 cm. **b**, Genotyping of the indicated plants by PCR and Sanger sequencing. LB-RP and LP-RP primer pairs specifically amplify mutated and non-mutated DNA, respectively.

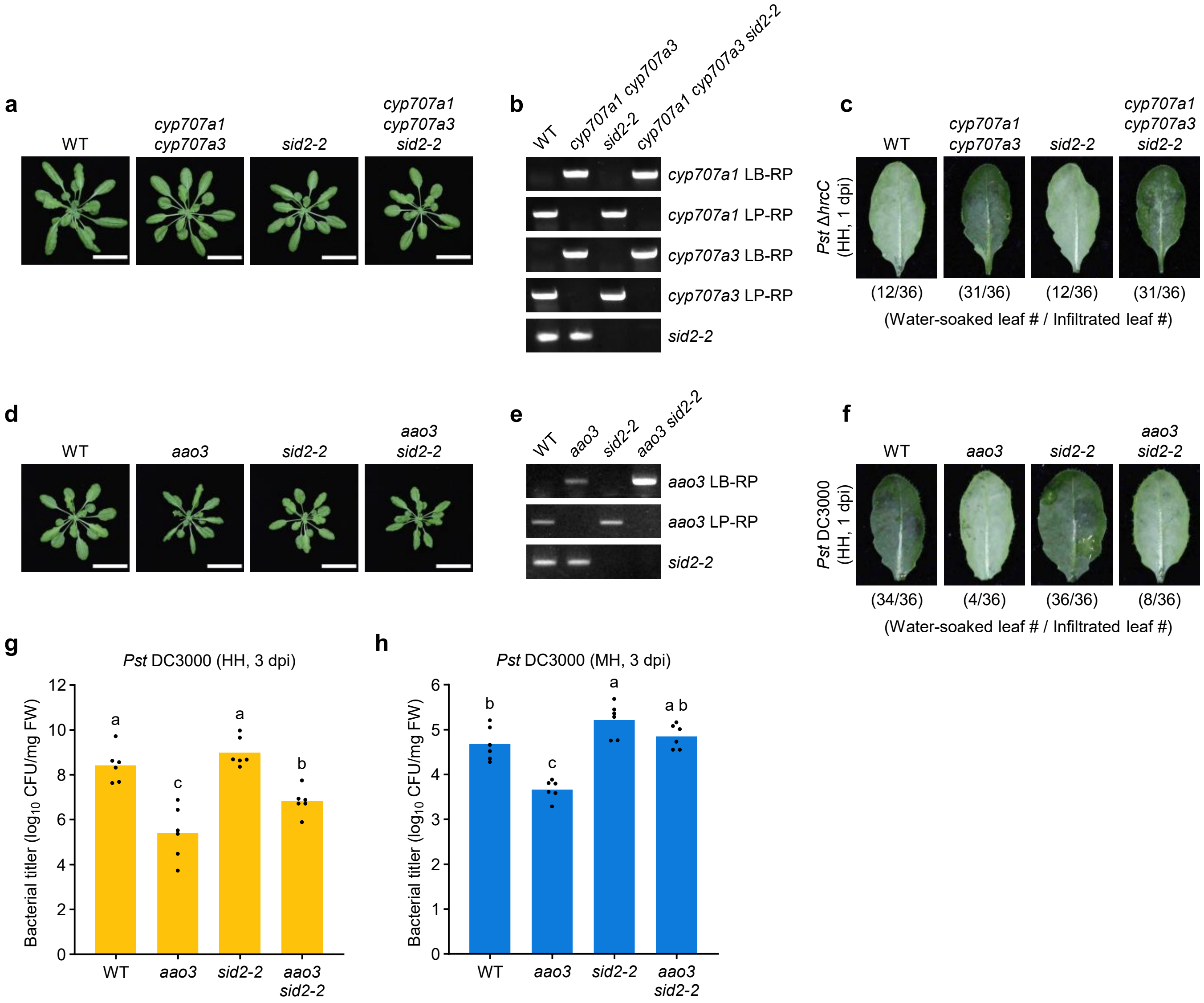

Supplementary Fig. 3: ABA depletion enhances bacterial resistance by restricting water-soaking independently of SA under high humidity.

a, **d**, Five-week-old plants grown under short-day conditions. Scale bars = 3 cm. **b**, **e**, Genotyping of the indicated plants by PCR. LB-RP and LP-RP primer pairs specifically amplify mutated and non-mutated DNA, respectively. **c**, **f**, Water-soaking in *Pst ΔhrcC*- (**c**) or *Pst* DC3000-inoculated (**f**) leaves. The indicated plants were infiltrated with *Pst ΔhrcC* or *Pst* DC3000 (OD_{600} = 0.02) and maintained under high humidity (HH) for 1 day. The number of water-soaked leaves was pooled from two independent experiments. **g**, **h**, Bacterial growth in *Pst* DC3000-inoculated leaves. The indicated plants were infiltrated with *Pst* DC3000 (OD_{600} = 0.0002) and maintained under HH (**g**) or moderate humidity (MH) (**h**) for 3 days. Bars represent means (n = 6 biological replicates). Different letters indicate statistically significant differences (P < 0.05; one-way ANOVA followed by Tukey's test). WT, wild-type; dpi, days post-infiltration. Experiments in (**g**, **h**) were repeated twice with similar results.

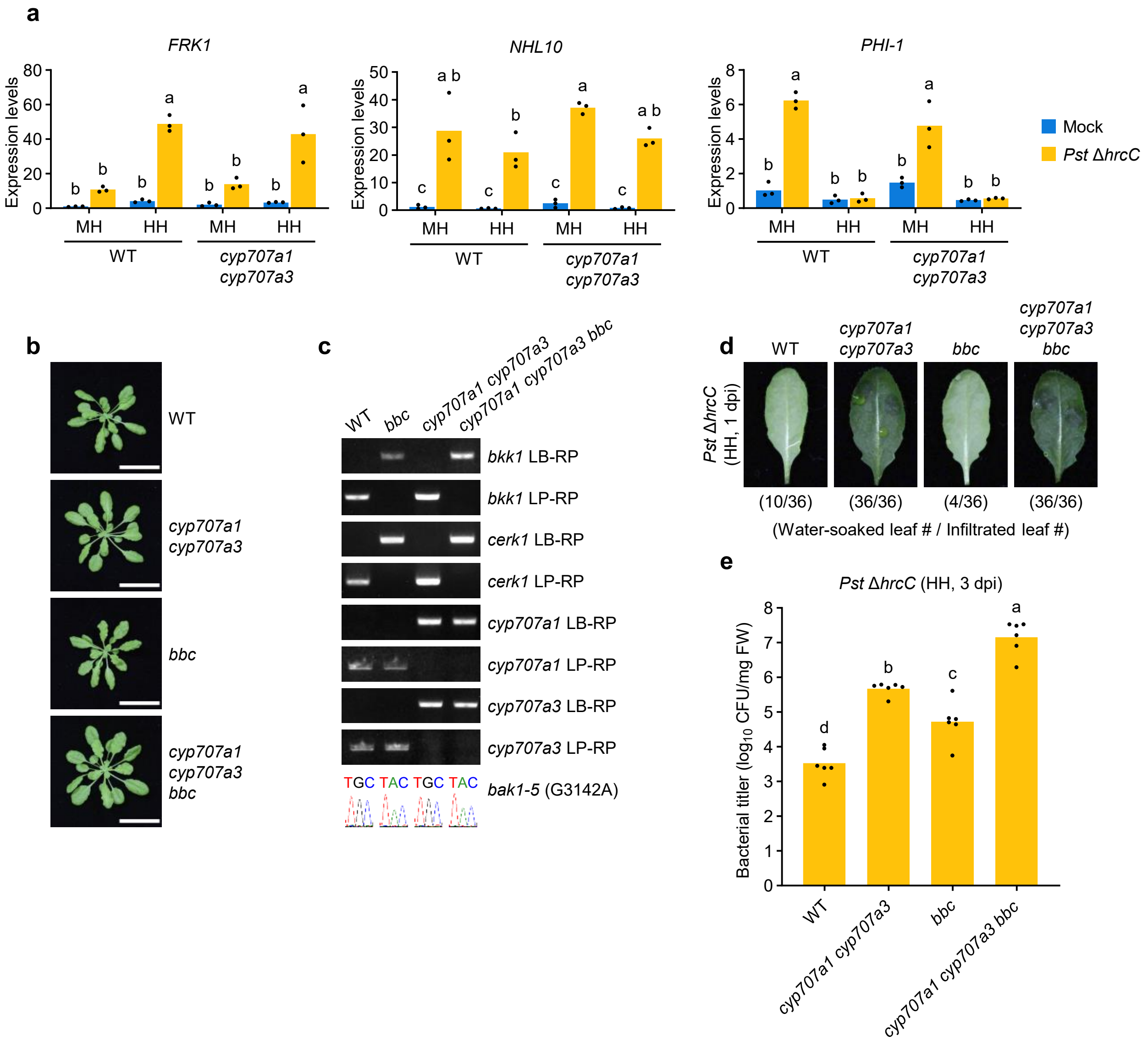

Supplementary Fig. 4: CYP707A3/A1-mediated ABA depletion and PTI contribute additively to bacterial resistance under high humidity.

a, Expression levels of *FRK1*, *NHL10*, and *PHI-1* in *Pst* Δ*hrcC*-infiltrated leaves under different humidity conditions. The indicated plants were maintained under moderate humidity (MH) or high humidity (HH) for 24 h before infiltration with water (Mock) or *Pst* Δ*hrcC* (OD_{600} = 0.2). Infiltrated plants were kept under the same humidity conditions for 1 h. Bars represent means (n = 3). **b**, Five-week-old plants grown under short-day conditions. Scale bars = 3 cm. **c**, Genotyping of the indicated plants by PCR and Sanger sequencing. LB-RP and LP-RP primer pairs specifically amplify mutated and non-mutated DNA, respectively. **d,** Water-soaking in *Pst* Δ*hrcC*-inoculated leaves. The indicated plants were infiltrated with *Pst* Δ*hrcC* (OD_{600} = 0.02) and maintained under HH for 1 day. The number of water-soaked leaves was pooled from two independent experiments. **e**, Bacterial growth in *Pst* Δ*hrcC*-inoculated leaves. The indicated plants were infiltrated with *Pst* Δ*hrcC* (OD_{600} = 0.02) and maintained under HH for 3 days. Bars represent means (n = 6). Sample size (n) indicates biological replicates. Different letters indicate statistically significant differences ($P < 0.05$; two-way ANOVA followed by Tukey's test in **a**; one-way ANOVA followed by Tukey's test in **e**). WT, wild-type; *bbc*, *bak1-5 bkk1 cerk1*; dpi, days post-infiltration. Experiments in (**a**, **e**) were repeated twice with similar results.

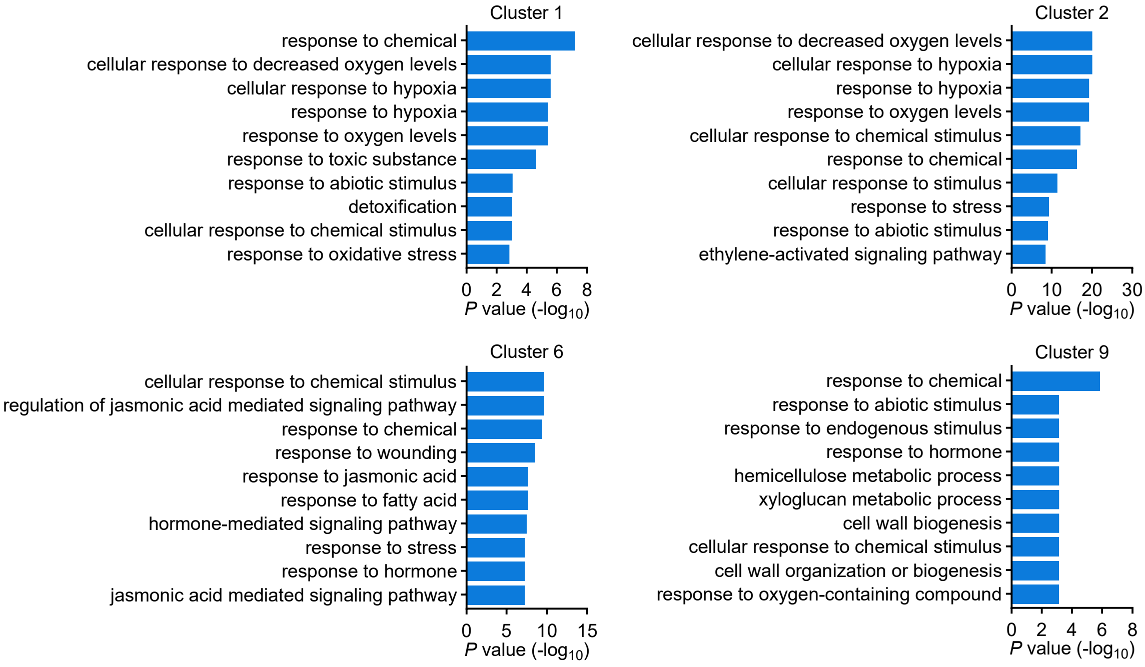

Supplementary Fig. 5: Gene Ontology analysis of high humidity-induced genes.
Top 10 enriched Gene Ontology categories of humidity-induced genes in the clusters identified in Fig. 3c. Enrichment significance was assessed using a two-sided hypergeometric test, and *P* values were adjusted for multiple comparisons using the Benjamini-Hochberg FDR method.

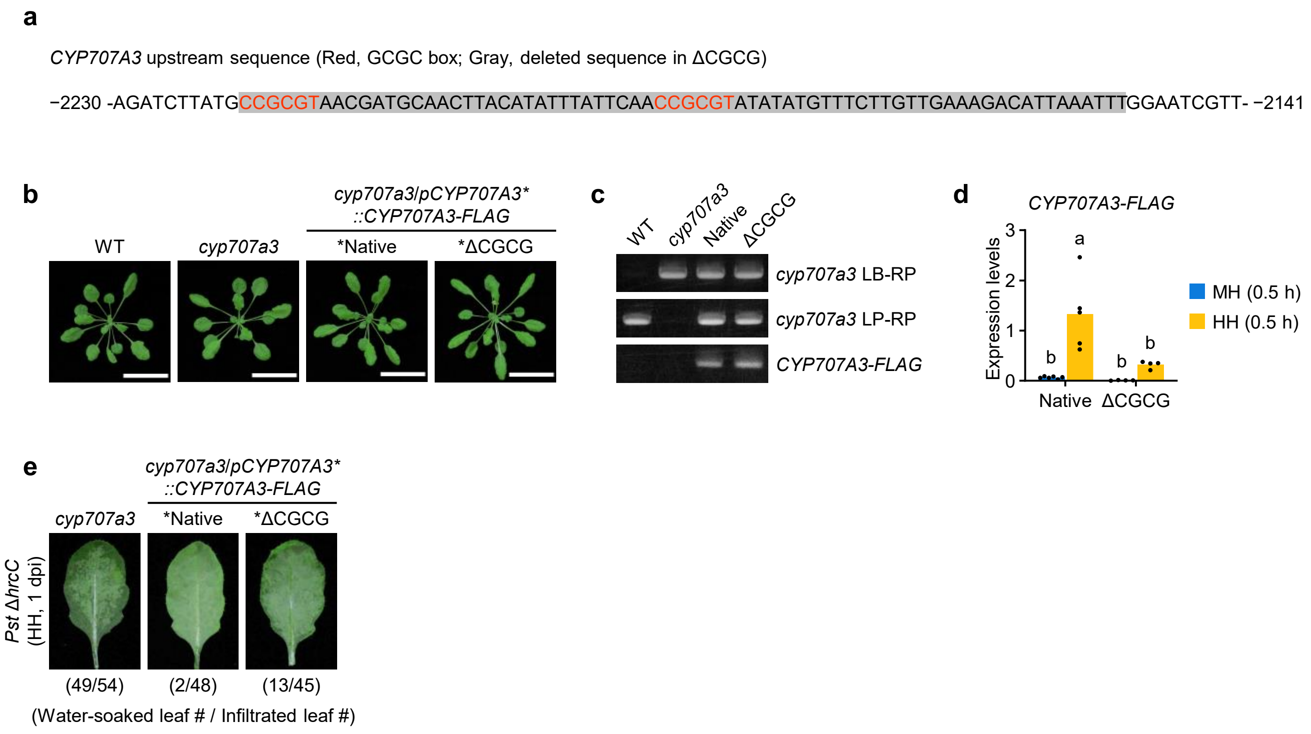

Supplementary Fig. 6: The CGCG boxes in the *CYP707A3* promoter are critical for water-soaking resistance.

a, Sequence of the *CYP707A*3 promoter region showing the deleted CGCG boxes in the ΔCGCG construct. Numbers indicate the position relative to the ATG start codon. **b**, Five-week-old plants grown under short-day conditions. Scale bars = 3 cm. **c**, Genotyping of the indicated plants by PCR. LB-RP and LP-RP primer pairs specifically amplify mutated and non-mutated DNA, respectively. **d**, Expression levels of *CYP707A3-FLAG* in humidity-treated leaves. The indicated plants were exposure to moderate humidity (MH) or high humidity (HH) for 0.5 h. Bars represent means (n = 3 biological replicates). Different letters indicate statistically significant differences ($P < 0.05$; two-way ANOVA followed by Tukey's test). **e**, Water-soaking in *Pst* Δ*hrcC*-inoculated leaves. The indicated plants were infiltrated with *Pst* Δ*hrcC* (OD_{600} = 0.02) and maintained under HH for 1 day. The number of water-soaked leaves was pooled from three independent experiments. WT, wild-type; dpi, days post-infiltration.

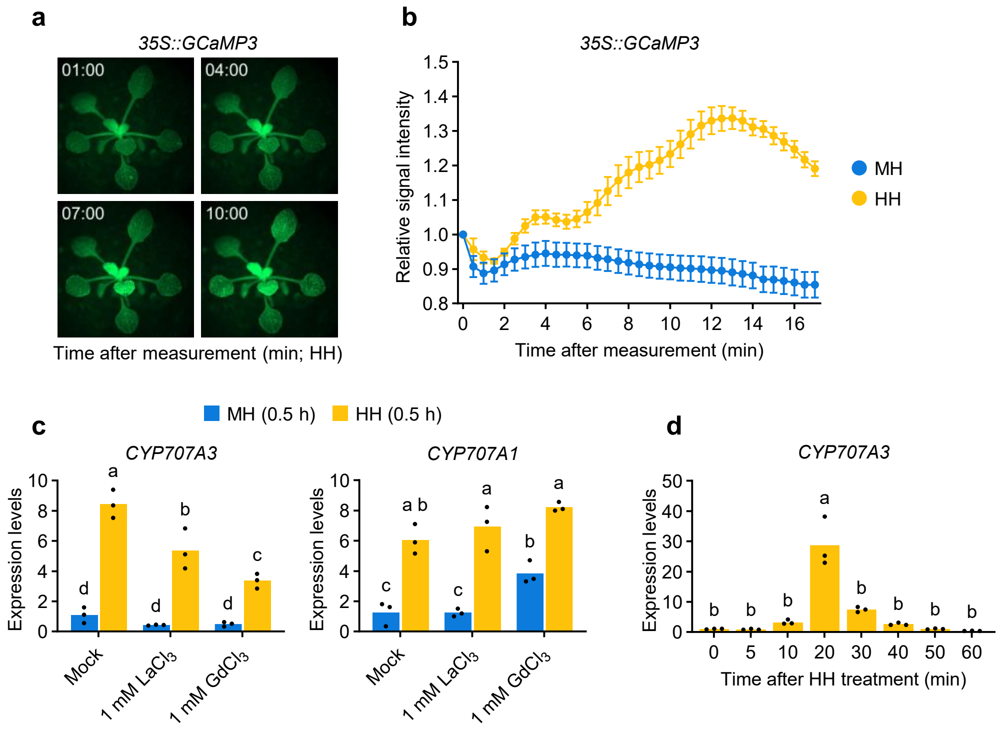

Supplementary Fig. 7: High humidity-triggered cytosolic Ca^{2+} elevation precedes *CYP707A3* induction.

a, **b,** Cytosolic Ca^{2+} levels in humidity-treated leaves. Two-week-old *35S::GCaMP3* transgenic plants grown under long-day conditions were exposed to moderate humidity (MH) or high humidity (HH) for the indicated time, and GCaMP3 fluorescence signals were measured. Representative GCaMP3 fluorescence images from HH-treated plants are shown in (**a**), and quantified GCaMP3 fluorescence signals are presented in (**b**). Bars represent means ± SEM (n = 10). **c**, Effect of Ca^{2+} channel inhibition on high humidity-induced *CYP707A3* and *CYP707A1* expression. Wild-type (WT) plants were infiltrated with water (Mock) or the indicated inhibitors, maintained under MH for 24 h, and then exposed to MH or HH for 0.5 h. Bars represent means (n = 3). **d**, Temporal dynamics of *CYP707A3* expression in high humidity-treated shoots. Two-week-old WT plants grown under long-day conditions were exposed to HH for the indicated time. Bars represent means (n = 3). Sample size (n) in (**b**) and (**c**, **d**) indicate leaves and biological replicates, respectively. Different letters indicate statistically significant differences ($P < 0.05$; two-way ANOVA followed by Tukey's test in **c**; one-way ANOVA followed by Tukey's test in **d**). Experiments in (**c**, **d**) were repeated twice with similar results.

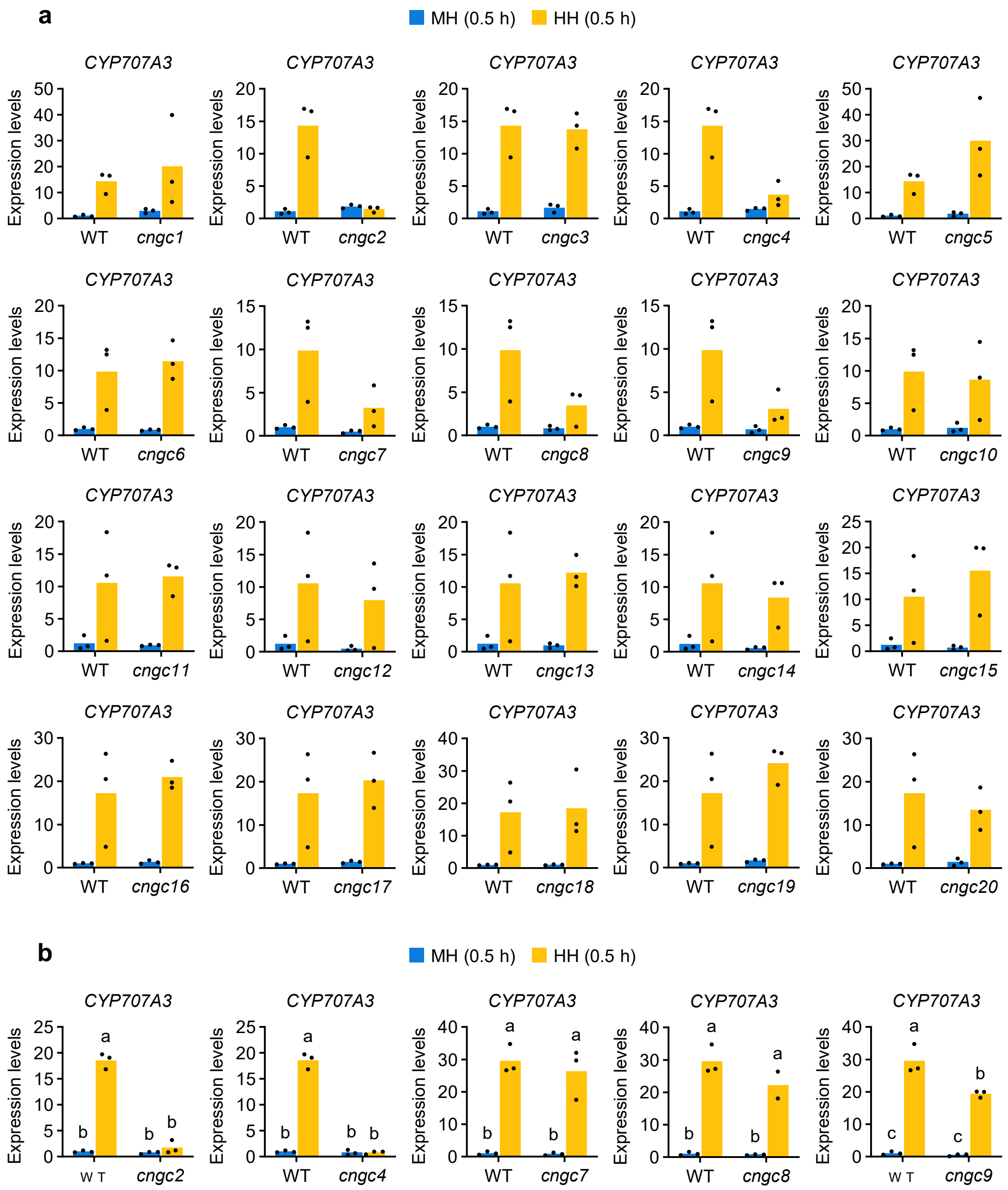

Supplementary Fig. 8: High humidity-induced *CYP707A3* expression is impaired in *cngc2*, *cngc4*, and *cngc9* mutant plants.

a, **b**, Expression levels of *CYP707A3* in humidity-treated shoots. The indicated plants were grown under long-day conditions for 2 weeks and then exposed to moderate humidity (MH) or high humidity (HH) for 0.5 h. Results from the first experiments are shown in (**a**), and results from the second experiments are shown in (**b**). Bars represent means (n = 3 biological replicates; *cngc8* treated with HH in **b**, n = 2). Different letters indicate statistically significant differences ($P < 0.05$; two-way ANOVA followed by Tukey's test). WT, wild-type.

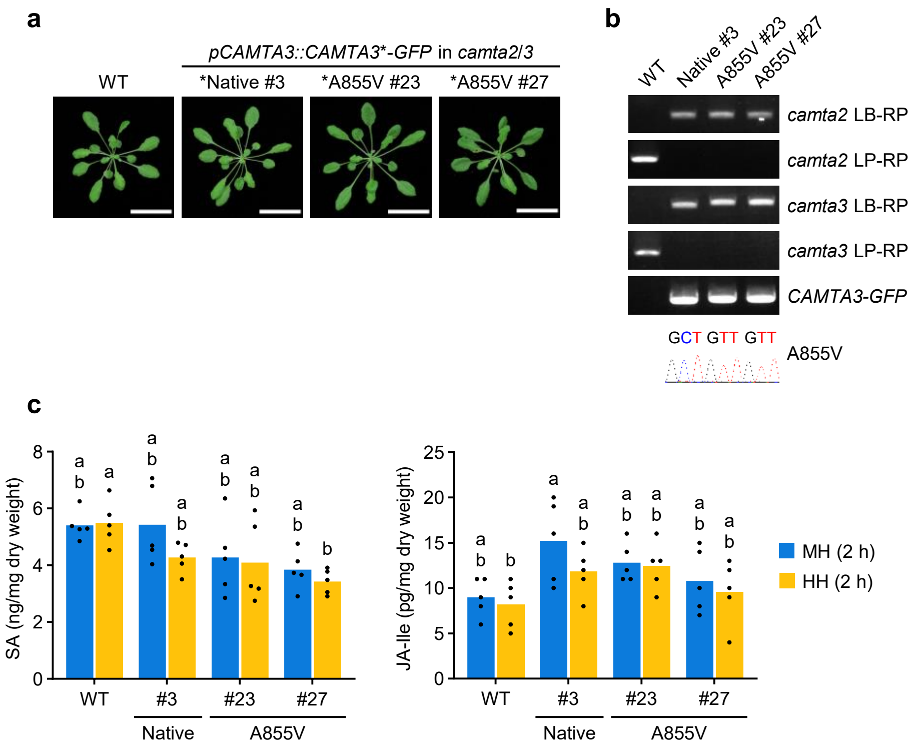

Supplementary Fig. 9: Characterization of *camta3* mutant plants.

a, Five-week-old plants grown under short-day conditions. Scale bars = 3 cm. **b**, Genotyping of the indicated plants by PCR and Sanger sequencing. LB-RP and LP-RP primer pairs specifically amplify mutated and non-mutated DNA, respectively. **c**, SA and JA-Ile levels in humidity-treated leaves. The indicated plants were exposed to moderate humidity (MH) or high humidity (HH) for 2 h. Bars represent means (n = 5 biological replicates). Different letters indicate statistically significant differences (P < 0.05; two-way ANOVA followed by Tukey's test). WT, wild-type; CAMTA3-A855V, a partial loss-of-function variant.

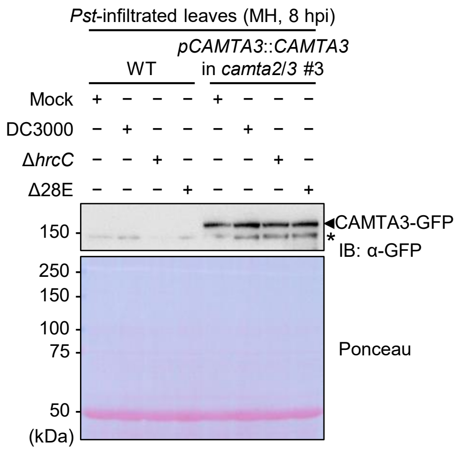

Supplementary Fig. 10: CAMTA3 protein levels in *Pst* DC3000-inoculated leaves.
Wild-type (WT) and transgenic plants expressing CAMTA3-GFP driven by the *CAMTA3* promoter (*pCAMTA3::CAMTA3* in *camta2/3* #3) were infiltrated with water (Mock) or the indicated *Pst* DC3000 strains (OD_{600} = 0.02) and maintained under moderate humidity (MH) for 8 h. CAMTA3-GFP was detected by immunoblotting with anti-GFP antibody (α-GFP). The ponceau-stained blot is shown as a loading control. The experiment was repeated three times with similar results.

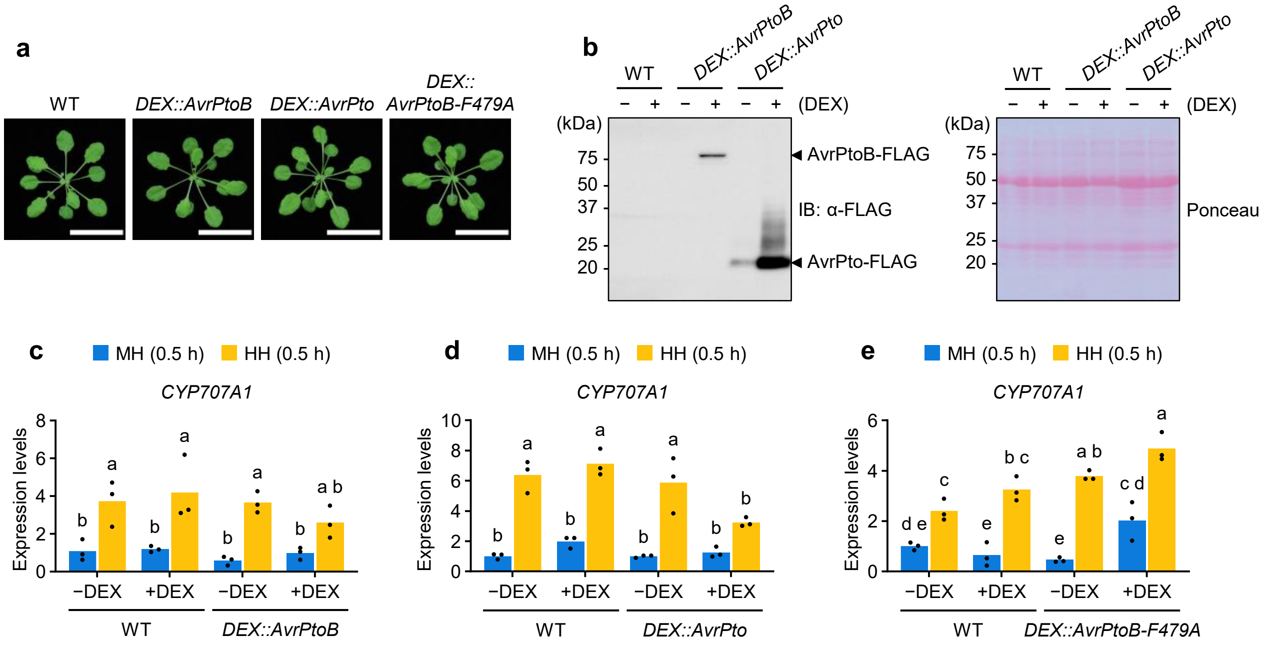

Supplementary Fig. 11: Characterization of *DEX::AvrPtoB*, *DEX::AvrPto*, *and DEX::AvrPtoB* transgenic plants.

a, Five-week-old plants grown under short-day conditions. Scale bars = 3 cm. **b**, Expression levels of AvrPtoB-FLAG and AvrPto-FLAG in DEX-treated leaves. The indicated plants were infiltrated with 0.1% ethanol (−DEX) or 10 µM DEX (+DEX) and maintained under moderate humidity (MH) for 24 h. AvrPtoB-FLAG and AvrPto-FLAG were detected by immunoblotting with anti-FLAG antibody (α-FLAG). The ponceau-stained blot is shown as a loading control. **c-e**, Expression levels of *CYP707A1* in humidity-treated leaves with or without *AvrPtoB* (**c**), *AvrPto* (**d**), and *AvrPtoB-F479A* (**e**). The indicated plants were treated with DEX as described in (**b**), followed by exposure to MH or high humidity (HH) for 0.5 h. Bars represent means (n = 3 biological replicates). Different letters indicate statistically significant differences ($P < 0.05$; two-way ANOVA followed by Tukey's test). The experiments in (**c**, **d**) were repeated twice with similar results.

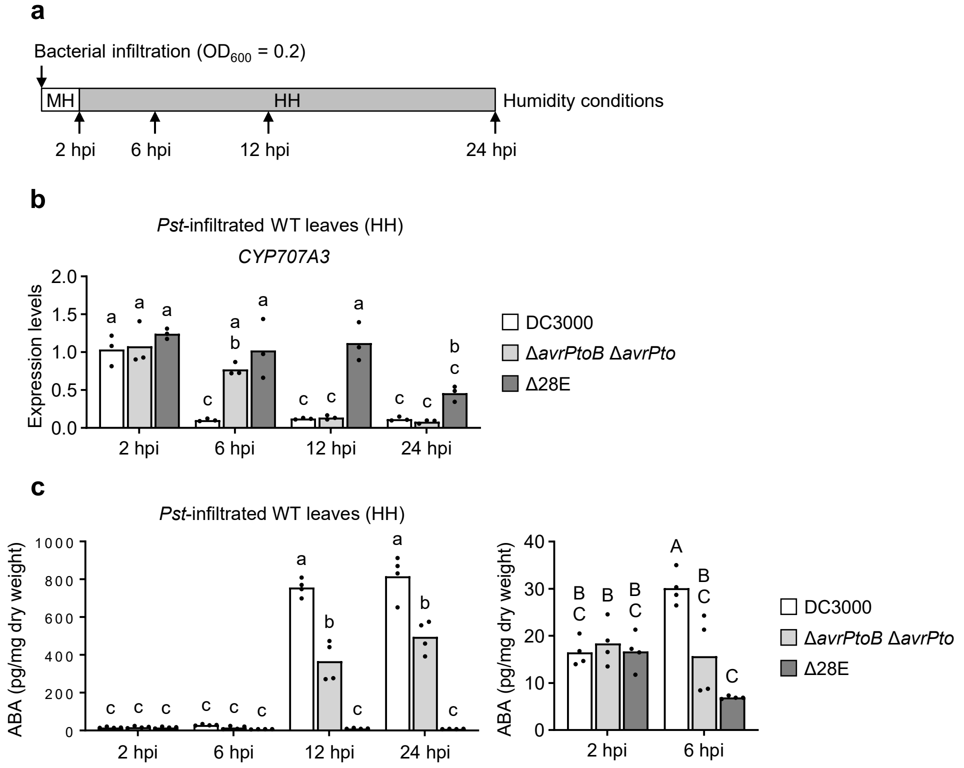

Supplementary Fig. 12: *Pst* DC3000-mediated *CYP707A3* suppression and ABA accumulation are compromised by loss of *avrPtoB* and *avrPto*

a, Schematic overview of the experimental design for monitoring temporal dynamics of *CYP707A3* expression and ABA accumulation in *Pst* DC3000-inoculated leaves under high humidity (HH). **b**, Temporal dynamics of *CYP707A3* expression in *Pst* DC3000-inoculated leaves. Wild-type (WT) plants were infiltrated with the indicated *Pst* DC3000 strains (OD_{600} = 0.2), maintained under moderate humidity (MH) for 2 h (2 hours post-infiltration, hpi), and then transferred to HH for 4 h (6 hpi), 10 h (12 hpi), or 22 h (24 hpi). Bars represent means (n = 3). **c**, Temporal dynamics of ABA levels in *Pst* DC3000-inoculated leaves. WT plants were infiltrated with the indicated *Pst* DC3000 strains as described in (**a**, **b**). Bars represent means (n = 4). Sample size (n) indicates biological replicates. Different letters indicate statistically significant differences ($P < 0.05$; two-way ANOVA followed by Tukey's test). Experiment in (**b**) was repeated twice with similar results.

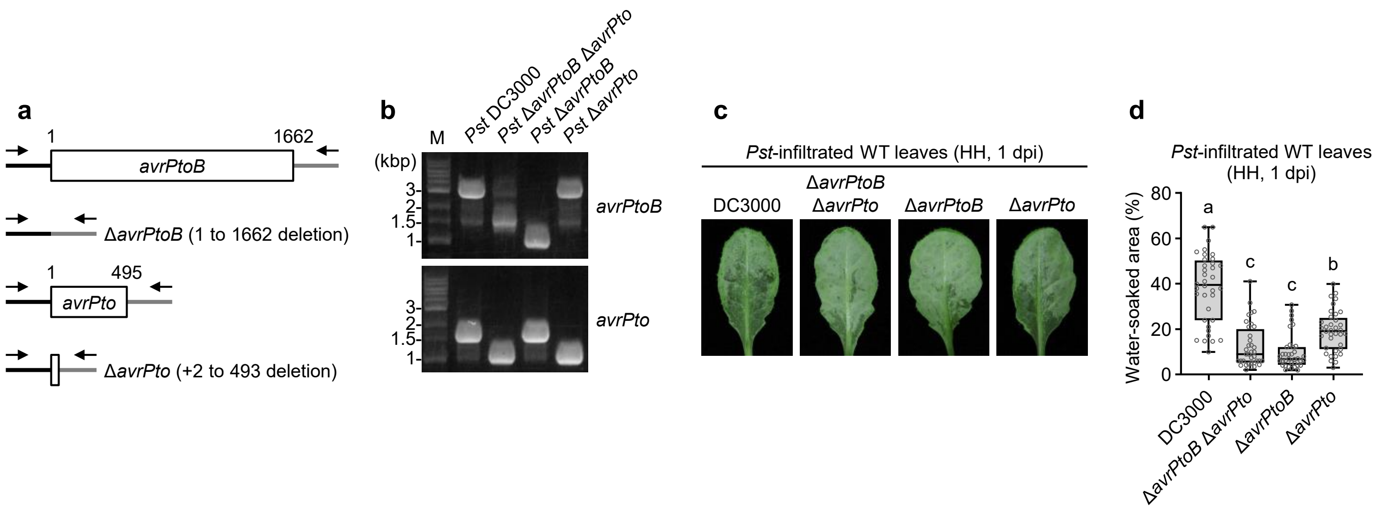

Supplementary Fig. 13: Characterization of *Pst* Δ*avrPtoB* and *Pst* Δ*avrPto* mutant strains.
a, b, Generation of *Pst* DC3000 mutant strains lacking *AvrPtoB* (Δ*avrPtoB*) or *AvrPto* (Δ*avrPto*). Deleted genomic regions (**a**) and genotyping results (**b**) of the established *Pst* DC3000 mutants. Numbers indicate the position relative to the ATG start codon. Arrows indicate genotyping primers. M, DNA size marker. **c**, **d**, Representative images (**c**) and quantification (**d**) of water-soaking in *Pst* DC3000-inoculated leaves. Wild-type (WT) plants were infiltrated with the indicated *Pst* DC3000 strains (OD_{600} = 0.2), maintained under MH for 2 h, and then transferred to HH for 20-22 h. Box plots represent the median, first and third quartiles, with whiskers showing the minimum and maximum values (n = 36 leaves; pooled from two independent experiments). Different letters indicate statistically significant differences (P < 0.05; one-way ANOVA followed by Tukey's test).
