# Supplementary material for: Humidity-driven ABA depletion determines plant-pathogen competition for leaf water
Source: Nat Commun. 2025 Dec 19;17:787. doi: 10.1038/s41467-025-67469-y (PMC12824301; doi:10.1038/s41467-025-67469-y)
Supplement: Supplementary file 2 — Description of Additional Supplementary Files [file 41467_2025_67469_MOESM2_ESM.docx]

**Supplementary Data 1**

Detailed information of DEGs in Fig. 3a.

**Supplementary Data 2**

Detailed information of 1000 most variable genes in Fig. 3c.

**Supplementary Data 3**

Detailed information of 2000 most variable genes in Fig. 5e.

**Supplementary Data 4**

List of Arabidopsis plants used in this study.

**Supplementary Data 5**

List of plasmid DNA used in this study.

**Supplementary Data 6:**

List of primers used in this study.

**Supplementary Data 7**

List of accession numbers related to this study.

**Supplementary Movie 1:**

Cytosolic Ca^2+^ dynamics in leaves upon exposure to high humidity. Arabidopsis transgenic plants expressing *35S::GCaMP3* were exposed to high humidity, and cytosolic Ca^2+^ changes were visualized through GCaMP3 fluorescence. Signals were captured at 0.08 min intervals over the indicated time course.
