# Supplementary material for: Humidity-driven ABA depletion determines plant-pathogen competition for leaf water
Source: Nat Commun. 2025 Dec 19;17:787. doi: 10.1038/s41467-025-67469-y (PMC12824301; doi:10.1038/s41467-025-67469-y)
Supplement: Supplementary file 11 — Reporting Summary [file 41467_2025_67469_MOESM11_ESM.pdf]

nature portfolio

Corresponding author(s): Shigetaka Yasuda
Yusuke Saijo

Last updated by author(s): Nov 28, 2025

Reporting Summary

Nature Portfolio wishes to improve the reproducibility of the work that we publish. This form provides structure for consistency and transparency in reporting. For further information on Nature Portfolio policies, see our Editorial Policies and the Editorial Policy Checklist.

Statistics

For all statistical analyses, confirm that the following items are present in the figure legend, table legend, main text, or Methods section.

n/a	Confirmed	
☐	☒	The exact sample size (n) for each experimental group/condition, given as a discrete number and unit of measurement
☐	☒	A statement on whether measurements were taken from distinct samples or whether the same sample was measured repeatedly
☐	☒	The statistical test(s) used AND whether they are one- or two-sided<br>*Only common tests should be described solely by name; describe more complex techniques in the Methods section.*
☒	☐	A description of all covariates tested
☐	☒	A description of any assumptions or corrections, such as tests of normality and adjustment for multiple comparisons
☐	☒	A full description of the statistical parameters including central tendency (e.g. means) or other basic estimates (e.g. regression coefficient) AND variation (e.g. standard deviation) or associated estimates of uncertainty (e.g. confidence intervals)
☐	☒	For null hypothesis testing, the test statistic (e.g. F, t, r) with confidence intervals, effect sizes, degrees of freedom and P value noted<br>*Give P values as exact values whenever suitable.*
☒	☐	For Bayesian analysis, information on the choice of priors and Markov chain Monte Carlo settings
☒	☐	For hierarchical and complex designs, identification of the appropriate level for tests and full reporting of outcomes
☒	☐	Estimates of effect sizes (e.g. Cohen's d, Pearson's r), indicating how they were calculated

Our web collection on statistics for biologists contains articles on many of the points above.

Software and code

Policy information about availability of computer code

Data collection	Agarose gel electrophoresis: FAS-BG LED BOX software (Nippon Genetics)<br>Sanger sequencing: 3500 Series Data Collection Software v3.3 (Applied Biosystems)<br>RT-qPCR: Thermal Cycler Dice Real Time System Ver. 6.01E for TP950 (TaKaRa)<br>Immunoblotting: FUSION-CAPT FOR FUSION FX7 EDGE, SOLO 7S EDGE 18.09 (Vilber)<br>Stomatal aperture measurement: Leica Application Suite X 3.4.2.18368 (Leica)<br>Real-time cytosolic Ca2+ imaging: ZEN Pro (ZEISS)
Data analysis	Image data: Image J Fiji (https://imagej.net/software/fiji/downloads); Adobe Photoshop Element 9.03<br>Sanger sequencing: BioEdit version 7.2.5 (https://bioedit.software.informer.com/7.2/)<br>Bacterial water-soaking quantification: Image J Fiji (https://imagej.net/software/fiji/downloads)<br>Phytohormone quantification: MassLynx4.2 (Waters)<br>Stomatal aperture measurement: Image J Fiji (https://imagej.net/software/fiji/downloads)<br>Real-time cytosolic Ca2+ imaging: ZEN Pro (ZEISS)<br>RNA-sequencing: CLC genomics workbench version 21 (Qiagen)<br>RNA-sequencing: iDEP 2.01 (https://bioinformatics.sdstate.edu/idep/)<br>RNA-sequencing: ShinyGO 0.81 (https://bioinformatics.sdstate.edu/go/)<br>Statistical analysis and graph creation: GraphPad Prism 10 (https://www.graphpad.com/features)

For manuscripts utilizing custom algorithms or software that are central to the research but not yet described in published literature, software must be made available to editors and reviewers. We strongly encourage code deposition in a community repository (e.g. GitHub). See the Nature Portfolio guidelines for submitting code & software for further information.

Data

Policy information about availability of data

All manuscripts must include a data availability statement. This statement should provide the following information, where applicable:

- Accession codes, unique identifiers, or web links for publicly available datasets
- A description of any restrictions on data availability
- For clinical datasets or third party data, please ensure that the statement adheres to our policy

All data generated and analyzed in this study are freely available without any restrictions. All raw data, including uncropped blots and qPCR Ct values underlying all figures, are provided in the Source Data file. The raw RNA sequencing data have been deposited in the DDJB database under accession code PRJDB17982 [https://ddbj.nig.ac.jp/search/entry/bioproject/PRJDB17982]

Research involving human participants, their data, or biological material

Policy information about studies with human participants or human data. See also policy information about sex, gender (identity/presentation), and sexual orientation and race, ethnicity and racism.

Reporting on sex and gender	N/A
Reporting on race, ethnicity, or other socially relevant groupings	N/A
Population characteristics	N/A
Recruitment	N/A
Ethics oversight	N/A

Note that full information on the approval of the study protocol must also be provided in the manuscript.

Field-specific reporting

Please select the one below that is the best fit for your research. If you are not sure, read the appropriate sections before making your selection.

☒ Life sciences ☐ Behavioural & social sciences ☐ Ecological, evolutionary & environmental sciences

For a reference copy of the document with all sections, see nature.com/documents/nr-reporting-summary-flat.pdf

Life sciences study design

All studies must disclose on these points even when the disclosure is negative.

Sample size	Sample size was determined based on experimental trials and prior publications on similar experiments. No statistical methods were used to predetermine sample size.
Data exclusions	No data were excluded from the anlayses.
Replication	The number of independent experiments is provided in the figure legends.
Randomization	No formal randomization was applied.
Blinding	Blinding was not applied, as experimental and control groups were clearly distinguishable by appearance and treatment.

Reporting for specific materials, systems and methods

We require information from authors about some types of materials, experimental systems and methods used in many studies. Here, indicate whether each material, system or method listed is relevant to your study. If you are not sure if a list item applies to your research, read the appropriate section before selecting a response.

Materials & experimental systems

n/a	Involved in the study	
☐	☒	Antibodies
☒	☐	Eukaryotic cell lines
☒	☐	Palaeontology and archaeology
☒	☐	Animals and other organisms
☒	☐	Clinical data
☒	☐	Dual use research of concern
☐	☒	Plants

Methods

n/a	Involved in the study	
☒	☐	ChIP-seq
☒	☐	Flow cytometry
☒	☐	MRI-based neuroimaging

Antibodies

Antibodies used

Anti-FLAG antibody (Sigma-Aldrich, Cat# F1804; 1:5000 dilution with TBS-T)
Anti-GFP antibody (MBL, Cat# 598 1:5000 dilution with TBS-T)
Anti-mouse IgG HRP-linked antibody (Cell Signaling Technology, Cat# 7076 1:5000 dilution with TBS-T)
Anti-rabbit IgG HRP-linked antibody (Cell Signaling Technology, Cat# 7074 1:5000 dilution with TBS-T)

Validation

The antibodies used in this study were validated by the supplier.

Dual use research of concern

Policy information about dual use research of concern

Hazards

Could the accidental, deliberate or reckless misuse of agents or technologies generated in the work, or the application of information presented in the manuscript, pose a threat to:

No	Yes	
☒	☐	Public health
☒	☐	National security
☒	☐	Crops and/or livestock
☒	☐	Ecosystems
☒	☐	Any other significant area

Experiments of concern

Does the work involve any of these experiments of concern:

No	Yes	
☒	☐	Demonstrate how to render a vaccine ineffective
☒	☐	Confer resistance to therapeutically useful antibiotics or antiviral agents
☒	☐	Enhance the virulence of a pathogen or render a nonpathogen virulent
☒	☐	Increase transmissibility of a pathogen
☒	☐	Alter the host range of a pathogen
☒	☐	Enable evasion of diagnostic/detection modalities
☒	☐	Enable the weaponization of a biological agent or toxin
☒	☐	Any other potentially harmful combination of experiments and agents

Plants

Seed stocks	Information on the sources and characteristics of the seeds used in this study is provided in the Methods section, Acknowledgments, and Supplementary Data 4.
Novel plant genotypes	The cyp707a1 cyp707a3 ost2-3D, cyp707a1 cyp707a3 sid2-2, aao3 sid2-2, bak1-5 bkk1 cerk1 (bbc), and cyp707a1 cyp707a3 bbc mutants were generated by genetic crossing. The cyp707a3/pCYP707A3::CYP707A3-FLAG, cyp707a3/pCYP707A3ΔCGCG::CYP707A3-FLAG, Col-0/DEX::AvrPtoB-FLAG, Col-0/DEX::AvrPto-FLAG, and Col-0/DEX::AvrPtoB-F479A-FLAG transgenic plants were generated by agrobacterium-mediated transformation.
Authentication	All seed stocks used in this study were verified for genotype by PCR and Sanger-sequencing.
